# 供患者性别组合对恶性血液病患者单倍体造血干细胞移植结局的影响

**DOI:** 10.3760/cma.j.issn.0253-2727.2022.12.004

**Published:** 2022-12

**Authors:** 姗姗 胡, 一波 吴, 盼盼 朱, 继敏 施, 建 余, 妍敏 赵, 晓瑜 来, 丽珍 刘, 华睿 傅, 河 黄, 依 罗

**Affiliations:** 1 浙江大学医学院附属第一医院骨髓移植中心，杭州 310000 Bone Marrow Transplantation Center, the First Affiliated Hospital, Zhejiang University School of Medicine, Hangzhou 310000, China; 2 浙江大学血液学研究所，杭州 310000 Institute of Hematology, Zhejiang University, Hangzhou 310000, China; 3 金华市人民医院血液科，金华 321000 Department of Hematology, Jinhua People's Hospital, Jinhua 321000, China

**Keywords:** 单倍体造血干细胞移植, 临床结局, 供者选择, Haploidentical hematopoietic stem cell transplantation, Clinical outcome, Haploidentical donor

## Abstract

**目的:**

探究供患者性别组合对基于抗胸腺细胞球蛋白（ATG）和单纯外周血干细胞（PBSC）来源单倍体造血干细胞移植（haplo-HSCT）治疗恶性血液病移植结局的影响。

**方法:**

对2015年2月至2020年9月在浙江大学医学院附属第一医院骨髓移植中心接受清髓性haplo-HSCT的648例恶性血液病患者进行回顾性分析。男363例（56.0％），女285例（44.0％），中位年龄32（14～62）岁。急性淋巴细胞白血病（ALL）242例（37.3％），急性髓系白血病（AML）293例（45.2％），骨髓增生异常综合征（MDS）56例（8.7％），非霍奇金淋巴瘤（NHL）27例（4.2％），其他恶性血液病30例（4.6％）。

**结果:**

①所有患者移植后3年总生存（OS）率为（73.10±1.90）％，无病生存（DFS）率为（70.80±1.90）％，Ⅱ~Ⅳ度、Ⅲ/Ⅳ度急性移植物抗宿主病（GVHD）累积发生率分别为（33.96±1.87）％、（13.08±1.33）％，3年中/重度慢性GVHD、重度慢性GVHD累积发生率分别为（35.10±2.14）％、（10.66±1.38）％，累积复发率（CIR）为（19.43±1.67）％，非复发死亡率（NRM）为（9.80±1.24）％。②供患者性别相合、性别不合组haplo-HSCT后28 d累积中性粒细胞植入率、血小板植入率差异无统计学意义（*P*＝0.270，*P*＝0.842），Ⅱ~Ⅳ度、Ⅲ/Ⅳ度急性GVHD累积发生率及移植后3年OS率、DFS率、CIR、NRM率、慢性GVHD累积发生率差异均无统计学意义（*P*>0.05）。③男供女、女供女、男供男、女供男组haplo-HSCT后28 d累积中性粒细胞植入率差异无统计学意义（*P*＝0.148），Ⅱ~Ⅳ度、Ⅲ/Ⅳ度急性GVHD累积发生率及移植后3年OS率、DFS率、CIR、NRM、慢性GVHD累积发生率差异均无统计学意义（*P*>0.05）。女供男组移植后28 d累积血小板植入率［（91.45±2.63）％］低于男供女组［（94.77±1.75）％，*P*＝0.004］、女供女组［（95.54±2.05）％，*P*＝0.005］，与男供男组［（95.08±1.41）％，*P*＝0.284］比较差异无统计学意义。④在≤35岁患者中，姐妹供者组、兄弟供者组移植后3年重度慢性GVHD累积发生率分别为（26.71±5.90）％、（10.33±4.43）％（*P*＝0.054）；在>35岁患者中，女儿供者组、儿子供者组移植后3年中重度慢性GVHD累积发生率分别为（40.07±6.65）％、（27.41±4.54）％（*P*＝0.084）。⑤女供男组移植后28 d累积血小板植入率低于其他组［（91.45±2.63）％对（95.08±0.95）％，*P*＝0.037］。⑥抗人T细胞兔免疫球蛋白10 mg/kg预防GVHD方案女供男组的28 d累积血小板植入率低于其他组［（89.29±4.29）％对（94.49±1.45）％，*P*＝0.037］，兔抗人胸腺细胞球蛋白6 mg/kg预防GVHD方案女供男组与其他组移植后28 d累积血小板植入率差异无统计学意义［（93.44±3.38）％对（95.62±1.26）％，*P*＝0.404］。

**结论:**

在基于ATG和PBSC来源的haplo-HSCT模式下，供患者性别组合对恶性血液病患者haplo-HSCT后主要临床结局没有影响。女供男模式与血小板植入延迟和较低的移植后28 d累积血小板植入率相关。

## Abstract (en)

**Objective:**

To investigate how gender differences between the donor and the recipient affect the effectiveness of antithymocyte globulin (ATG) and pure peripheral blood stem cell (PBSC) hematopoietic stem cell transplantation (haplo-HSCT) in the treatment of malignant hematological diseases.

**Methods:**

From February 2015 to September 2020, 648 hematological malignancies patients underwent myeloablative condition regimen haplo-HSCT treatment at the Bone Marrow Transplant Center of the First Affiliated Hospital of Zhejiang University. The median age was 32 (14–62) years, with 363 males (56.0％) and 285 females (44.0％) present. 242 cases of acute lymphoblastic leukemia（ALL）（37.3％）, 293 cases of acute myeloid leukemia（AML）（45.2％）, 56 cases of myelodysplastic syndrome（MDS）（8.7％）, 27 cases of non-Hodgkin's lymphoma（NHL）（4.2％）, and 30 cases of other hematological malignancies（4.6％）.

**Results:**

① The 3-year overall survival（OS）, DFS, the incidence of Ⅱ–Ⅳ grade acute graft-versus-host disease（aGVHD）, the incidence of Ⅲ–Ⅳ grade aGVHD, the 3-year incidence of moderate & severe chronic GVHD（cGVHD）, severe cGVHD, the 3-year incidence of relapse, and NRM of the whole group were（73.10±1.90）％,（70.80±1.90）％,（33.96±1.87）％,（13.08±1.33）％,（35.10±2.14）％,（10.66±1.38）％,（19.43±1.67）％, and（9.80±1.24）％, respectively. ②There was no statistically significant difference between the donor-recipient gender match and donor-recipient gender mismatch groups in the 28-day cumulative neutrophil engraftment rate, 28-day cumulative platelet engraftment rate, the incidence of Ⅱ–Ⅳ grade aGVHD, the incidence of Ⅲ–Ⅳ grade aGVHD, 3-year OS, 3-year DFS, the cumulative incidence of relapse, NRM, and incidence of moderate & severe cGVHD, severe cGVHD. ③The 28-day cumulative neutrophil engraftment rate did not differ statistically between the male-female, female-female, male-male, and female-male groups（*P*＝0.148）. The incidence of Ⅱ–Ⅳ grade aGVHD, the incidence of Ⅲ–Ⅳ grade aGVHD, 3-year OS, 3-year DFS, cumulative relapse rate, and NRM, and the incidence of cGVHD were not statistically different among the four groups（*P*>0.05）. The 28-day cumulative platelet engraftment rate of the female-male group was significantly lower than male-female group, and the female-female group［（91.45±2.63）％ *vs*.（94.77±1.75）％, *P*＝0.004;（91.45±2.63）％ *vs*.（95.54±2.05）％, *P*＝0.005］. No significant difference existed in the 28-day cumulative platelet engraftment rate between the female-male group and the male-male group［（91.45±2.63）％ *vs*.（95.08±1.41）％, *P*＝0.284］. ④Among patients ≤35 years old, the 3-year incidence of severe cGVHD patients receiving sister donors and sibling donors were（26.71±5.90）％ and（10.33±4.43）％, respectively（*P*＝0.054）. Patients accepting daughter donors and son donors had a 3-year incidence of moderate and severe cGVHD that was 40.07％ *vs*. 27.41％, respectively, among those over 35（40.07±6.65）％ *vs*.（27.41±4.54）％（*P*＝0.084）. ⑤Female donors to male recipients had a significantly lower 28-day cumulative platelet engraftment rate compared to the other groups［（91.45±2.63）％ *vs*.（95.08±0.95）％, *P*＝0.037］. ⑥ Female donors to male recipients had a significantly lower 28-day cumulative platelet engraftment rate than the other groups in the ATG-Fresenius（ATG-F）10 mg/kg group［（89.29±4.29）％ *vs*.（94.49±1.45）％, *P*＝0.037］. But when compared to the other groups in the Rabbit Antihuman Thymocyte Immunoglobulin（rATG-T）6 mg/kg group, the 28-day cumulative platelet implantation rate between female donors and male recipients was not significantly different［（93.44±3.38）％ *vs*.（95.62±1.26）％, *P*＝0.404］.

**Conclusion:**

The main clinical outcomes of patients with malignant blood diseases following transplantation are unaffected by the gender combination of the donor and patient in the haplo-HSCT mode based on ATG and PBSC sources. Female donors to male recipients have a lower 28-day cumulative platelet engraftment rate and longer platelet engraftment times.

异基因造血干细胞移植（allo-HSCT）是根治恶性血液病的主要方法[1]。近年来单倍体造血干细胞移植（haplo-HSCT）已取得与亲缘全相合、非血缘全相合造血干细胞移植相似的疗效，并已成为我国allo-HSCT的主要模式[2]–[4]。虽然患者相关因素如疾病、危险分层等是决定haplo-HSCT预后的主要因素，但已有多个研究证实供者相关因素也会影响haplo-HSCT的临床结局[5]–[7]。几乎每一个患者都有一个及以上的单倍体相合供者，如何选择单倍体相合供者提高移植疗效是目前临床急需解决的问题。既往研究显示，供患者性别组合可能会对haplo-HSCT的结局产生影响。Y染色体上存在次要组织相容性抗原、以及女性供者存在抗胎儿抗体等因素使得女供男模式下移植物抗宿主病（GVHD）发生率和非复发死亡率（NRM）增高[8]–[9]。此前在基于抗胸腺细胞球蛋白（ATG）和移植后环磷酰胺（PTCy）预防GVHD的体内非去T细胞（TCR）haplo-HSCT体系中均有报道显示女供男模式对患者的移植后生存具有不良影响[7],[10]–[15]。但在基于PTCy预防GVHD和体外去T细胞haplo-HSCT的临床研究中，也有供者性别并不影响移植结局的报道[16]，甚至显示母亲供者或女性供者与较低的累积复发率（CIR）和较高的总生存（OS）率相关[17]–[18]。因此供患者性别组合对haplo-HSCT结局的影响目前仍存在争议。本研究中，我们对2015年2月至2020年9月在本中心接受清髓性haplo-HSCT的恶性血液病患者进行回顾性分析，旨在探究在基于单纯PBSC来源和ATG预防GVHD的haplo-HSCT体系中供患者性别组合对恶性血液病移植结局的影响。

## 病例与方法

一、病例选择

本研究对2015年2月至2020年9月在浙江大学医学院附属第一医院骨髓移植中心接受清髓性haplo-HSCT的648例恶性血液病患者进行回顾性分析。所有患者均为首次接受allo-HSCT。移植前对所有患者和供者进行HLA-A、-B、-C、-DRB1和-DQB1的高分辨DNA分型检测。本研究经浙江大学医学院附属第一医院伦理委员会批准（2021-T729）。所有纳入研究的患者及其供者均在移植前签署知情同意书。

二、预处理方案

本组病例均采用清髓性预处理方案：阿糖胞苷4 g·m^−2^·d^−1^静脉滴注，−10 d、−9 d；白消安3.2 mg·kg^−1^·d^−1^静脉滴注，−8 d ～−6 d；环磷酰胺1.8 g·m^−2^·d^−1^静脉滴注，−5 d、−4 d，司莫司汀250 mg/m^2^口服，−3 d。

三、造血干细胞的动员和采集

所有供者均以粒细胞集落刺激因子（G-CSF）行造血干细胞动员（5～7.5 µg·kg^−1^·d^−1^，连续4～5 d）后进行外周血干细胞采集。所有移植物均未行接受体外去除T淋巴细胞处理。

四、GVHD预防

GVHD的预防采用霉酚酸酯、环孢素A加短程甲氨蝶呤联合ATG的方案，ATG包括抗人T细胞兔免疫球蛋白（ATG-F）2.5 mg·kg^−1^·d^−1^或兔抗人胸腺细胞免疫球蛋白（rATG-T）1.5 mg·kg^−1^·d^−1^，均为−5 d～−2 d静脉滴注给药，具体方案参见国内相关指南[19]–[20]和本中心既往报道[3],[21]。移植后6～9个月后无GVHD症状者减停免疫抑制剂。

五、随访

采用查阅门诊/住院病历和电话随访方式获得患者生存资料。随访截止日期为2021年2月28日。

六、定义

患者的危险分层根据2014年建立的疾病危险指数（rDRI）[22]分为低危、中危、高危、很高危。

中性粒细胞植入时间定义为连续3 d外周血中性粒细胞绝对计数（ANC）>0.5×10^9^/L，血小板植入定义为外周血PLT>20×10^9^/L连续7 d且脱离血小板输注。造血干细胞成功植入的患者被纳入急性GVHD（aGVHD）分析。aGVHD的诊断和分级依据2016年建立的Harri标准[23]。移植后存活超过100 d并且没有被排除aGVHD分析的患者纳入慢性GVHD（cGVHD）分析，cGVHD根据2014年美国国立卫生院（NIH）建立的cGVHD的诊断和分级标准[24]分为无、轻、中、重度。

OS期定义为从造血干细胞回输开始至各种原因导致死亡或随访终止的时间。无病生存（DFS）期定义为从造血干细胞回输开始至原发病复发或各种原因所致死亡或随访终止的时间。NRM定义为从造血干细胞回输开始至除原发病复发以外各种原因导致的死亡。复发定义为骨髓中原始细胞比例>5％或血液中再现原始细胞或出现髓外原始细胞浸润。

七、主要观察终点和次要观察终点

主要观察终点为移植后3年OS，次要观察终点包括移植后28 d中性粒细胞和血小板植入率、移植后100 d aGVHD累积发生率及移植后3年NRM、DFS、CIR、cGVHD累积发生率。

八、统计学处理

采用SPSS 26.0软件进行数据分析。对连续性变量进行正态分布检验，并根据检验结果进一步选择统计学方法。所有符合正态分布的连续性变量采用“均数±标准差”表示，不符合正态分布的连续性变量用“中位数（范围）”表示，分类变量用“频数”、“百分率”表示。基线表格组间比较计量资料比较采用Kruskal-Wallis *H*检验，计数资料比较采用*χ*^2^检验。OS、DFS采用Kaplan-Meier法描述其状态分布，并用Log-rank检验进行单因素预后分析，Cox回归模型进行多因素分析。采用*R* 3.6.1软件对中性粒细胞植入率、血小板植入率、GVHD发生率、CIR及NRM应用竞争风险分析评估累积发生率。应用Fine and Grey模型对Ⅱ～Ⅳ度aGVHD、CIR、NRM进行多因素分析，*P*<0.05为差异具有统计学意义。

## 结果

一、一般资料

本研究纳入648例连续性接受清髓性haplo-HSCT的恶性血液病患者，男363例（56.0％），女285例（44.0％），中位年龄32（14～62）岁。急性淋巴细胞白血病（ALL）242例（37.3％），急性髓系白血病（AML）293例（45.2％），骨髓增生异常综合征（MDS）56例（8.7％），非霍奇金淋巴瘤（NHL）27例（4.2％），其他恶性血液病30例（4.6％）。rDRI危险分层：低危/中危433例（66.8％），高危/很高危215例（33.2％）。ATG-F（总量10 mg/kg）组313例（48.3％），rATG-T（总量6 mg/kg）组335例（51.7％）。中位随访时间948（163～2196）d。不同供患者性别组合患者的一般及临床资料详见表1。

**表1:** 接受不同供患者性别组合单倍体造血干细胞移植恶性血液病患者的基本临床资料

临床指标	男供女（173例）	女供女（112例）	男供男（246例）	女供男（117例）	统计量	*P*值
患者年龄［岁，*M*（范围）］	33（14~62）	33.5（15~61）	30（14~61）	30（14~62）	*H*=4.427	0.219
疾病诊断［例（%）］					*χ*^2^=17.240	0.141
AML	75（43.4）	55（49.1）	108（43.9）	55（47.0）		
ALL	68（39.3）	45（40.2）	86（35.0）	43（36.8）		
MDS	21（12.1）	7（6.2）	20（8.1）	8（6.8）		
NHL	5（2.9）	1（0.9）	17（6.9）	4（3.4）		
其他	4（2.3）	4（3.6）	15（6.1）	7（6.0）		
rDRI危险分层［例（%）］					*χ*^2^=8.156	0.519
低危	5（2.9）	5（4.5）	19（7.7）	6（5.1）		
中危	103（59.5）	74（66.1）	151（61.4）	70（59.8）		
高危	55（31.8）	26（23.2）	62（25.2）	32（27.4）		
很高危	10（5.8）	7（6.2）	14（5.7）	9（7.7）		
回输MNC［×10^8^/kg，*M*（范围）］	16.00（2.08~55.28）	13.82（2.97~45.76）	14.65（2.34~44.80）	12.49（1.19~43.74）	*H*=16.083	0.001
回输CD34^+^细胞［×10^6^/kg，*M*（范围）］	6.69（1.75~28.7）	5.71（0.27~17.41）	5.80（1.31~22.87）	4.78（1.02~16.17）	*H*=30.805	<0.001
ATG种类［例（%）］					*χ*^2^=5.696	0.127
rATG-T 6 mg/kg	90（52.0）	68（60.7）	116（47.2）	61（52.1）		
ATG-F 10 mg/kg	83（48.0）	44（39.3）	130（52.8）	56（47.9）		

注 AML：急性髓系白血病；ALL：急性淋巴细胞白血病；MDS：骨髓增生异常综合征；NHL：非霍奇金淋巴瘤；rDRI：疾病危险指数；MNC：单个核细胞；ATG：抗胸腺细胞球蛋白；ATG-F：抗人T细胞兔免疫球蛋白；rATG-T：兔抗人胸腺细胞免疫球蛋白

二、整体移植结局

所有患者移植后3年OS率为（73.10±1.90）％，DFS率为（70.80±1.90）％，CIR为（19.43±1.67）％，NRM为（9.80±1.24）％。Ⅱ～Ⅳ度、Ⅲ/Ⅳ度aGVHD累积发生率分别为（33.96±1.87）％、（13.08±1.33）％，3年中重度cGVHD、重度cGVHD累积发生率分别为（35.10±2.14）％、（10.66±1.38）％。生存曲线见图1。

**图1:**
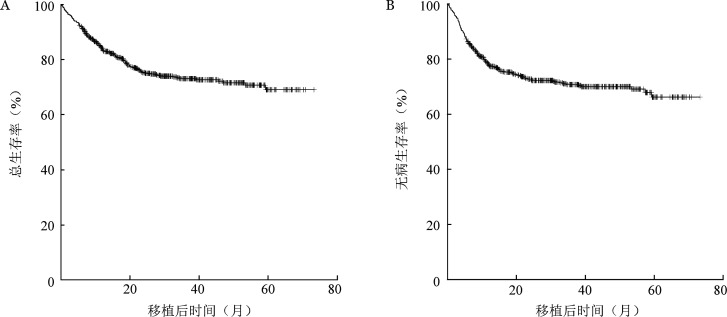
648例恶性血液病患者清髓性单倍体造血干细胞移植后总生存曲线（A）和无病生存曲线（B）

三、供患者不同性别组合模式对移植结局的影响

1. 供患者性别相合和性别不合组间比较：供患者性别相合、性别不合组haplo-HSCT后28 d累积中性粒细胞植入率、血小板植入率差异无统计学意义（*P*＝0.270，*P*＝0.842），Ⅱ～Ⅳ度、Ⅲ/Ⅳ度aGVHD累积发生率及移植后3年OS率、DFS率、CIR、NRM及cGVHD累积发生率差异均无统计学意义（*P*>0.05），详见表2。

**表2:** 供患者性别相合、不合恶性血液病患者haplo-HSCT后28 d造血重建及移植后3年主要临床结局［％，*x±s*）］

组别	例数	移植后28 d造血重建		GVHD累积发生率				CIR	NRM	OS率	DFS率
		中性粒细胞植入	血小板植入	Ⅱ~Ⅳ度aGVHD	Ⅲ/Ⅳ度aGVHD	中/重度cGVHD	重度cGVHD				
相合	358	99.16±0.50	95.22±1.14	35.67±2.54	13.48±1.81	37.48±2.94	11.60±1.91	20.24±2.30	9.31±1.67	73.40±2.60	70.50±2.60
不合	290	98.62±0.77	93.43±1.47	31.82±2.76	12.59±1.97	32.19±3.11	9.45±1.99	18.43±2.43	10.40±1.85	72.60±2.80	71.20±2.80

*χ*^2^值		1.219	0.040	1.012	0.107	2.389	1.156	0.251	0.265	0.033	<0.001
*P*值		0.270	0.842	0.315	0.744	0.122	0.282	0.616	0.607	0.856	0.998

注：haplo-HSCT：单倍体造血干细胞移植；GVHD：移植物抗宿主病，aGVHD、cGVHD分别为急、慢性GVHD；CIR：累积复发率；NRM：非复发死亡率；OS：总生存；DFS：无病生存

2. 男供女、女供女、男供男、女供男组间比较：男供女、女供女、男供男、女供男组haplo-HSCT后28 d累积中性粒细胞植入率差异无统计学意义（*P*＝0.148），Ⅱ～Ⅳ度、Ⅲ/Ⅳ度aGVHD累积发生率及移植后3年OS率、DFS率、CIR、NRM及cGVHD累积发生率差异均无统计学意义（*P*>0.05）。女供男组移植后28 d累积血小板植入率低于男供女组［（91.45±2.63）％对（94.77±1.75）％，*P*＝0.004］、女供女组［（91.45±2.63）％对（95.54±2.05）％，*P*＝0.005］，与男供男组比较差异无统计学意义［（91.45±2.63）％对（95.08±1.41）％，*P*＝0.284］。详见表3。

**表3:** 供患者不同性别组合模式恶性血液病患者haplo-HSCT后28 d造血重建及主要3年临床结局［％，*x±s*）］

组别	例数	移植后28 d造血重建		GVHD累积发生率				CIR	NRM	OS率	DFS率
		中性粒细胞植入	血小板植入	Ⅱ~Ⅳ度aGVHD	Ⅲ/Ⅳ度aGVHD	中、重度cGVHD	重度cGVHD				
男供女	173	98.84±1.01	94.77±1.75	31.58±3.57	14.04±2.66	32.78±4.10	7.83±2.41	18.60±3.20	10.24±2.38	73.60±3.60	71.20±3.70
女供女	112	100.00±0.89	95.54±2.05	35.71±4.55	14.29±3.32	41.91±5.40	18.63±4.45	19.02±4.20	8.87±3.20	76.10±4.80	72.10±4.90
男供男	246	98.77±0.73	95.08±1.41	35.66±3.07	13.12±2.17	35.31±3.49	8.62±1.96	20.83±2.77	9.61±1.99	72.00±3.10	69.60±3.10
女供男	117	96.58±1.71	91.45±2.63	32.17±4.38	10.44±2.86	31.36±4.82	11.60±3.38	18.40±3.79	10.66±2.95	71.20±4.50	70.90±4.40

*χ*^2^值		5.353	14.116	1.013	0.986	4.080	7.381	1.080	0.787	1.705	1.649
*P*值		0.148	0.003	0.798	0.805	0.253	0.061	0.782	0.853	0.636	0.648

注：haplo-HSCT：单倍体造血干细胞移植；GVHD：移植物抗宿主病，aGVHD、cGVHD分别为急、慢性GVHD；CIR：累积复发率；NRM：非复发死亡率；OS：总生存；DFS：无病生存

3. 女供男组与其他供患者性别组组间比较：女供男组与其他组haplo-HSCT后28 d累积中性粒细胞植入率差异无统计学意义（*P*＝0.554），Ⅱ～Ⅳ度、Ⅲ/Ⅳ度aGVHD累积发生率及移植后3年OS率、DFS、CIR率、NRM、cGVHD累积发生率差异均无统计学意义（*P*>0.05）。女供男组移植后28 d累积血小板植入率显著低于其他组［（91.45±2.63）％对（95.08±0.95）％，*P*＝0.037］，详见表4。将患者的年龄、供者年龄、rDRI及接受不同剂量/类型ATG、女供男/其他纳入多因素分析，结果发现女供男不影响Ⅱ～Ⅳ度、Ⅲ/Ⅳ度aGVHD累积发生率3年OS、DFS、CIR、NRM及移植后3年中重度、重度cGVHD累积发生率（*P*>0.05），详见表5。

**表4:** 女供男组合与其他供患者性别组合恶性血液病患者haplo-HSCT后28 d造血重建及主要3年临床结局［％，*x±s*）］

组别	例数	移植后28 d造血重建		GVHD累积发生率				CIR	NRM	OS率	DFS率
		中性粒细胞植入	血小板植入	Ⅱ~Ⅳ度aGVHD	Ⅲ/Ⅳ度aGVHD	中、重度cGVHD	重度cGVHD				
女供男	117	96.58±1.71	91.45±2.63	32.17±4.38	10.44±2.86	31.36±4.82	11.60±3.38	18.40±3.79	10.66±2.95	71.20±4.50	70.90±4.40
其他	531	99.24±0.42	95.08±0.95	34.35±2.07	13.66±1.50	35.92±2.38	10.40±1.51	19.70±1.87	9.62±1.37	73.40±2.10	70.70±2.10

*χ*^2^值		0.351	4.357	0.248	0.874	0.641	0.056	0.036	0.101	0.042	0.007
*P*值		0.554	0.037	0.618	0.350	0.423	0.814	0.850	0.750	0.838	0.932

注：haplo-HSCT：单倍体造血干细胞移植；GVHD：移植物抗宿主病，aGVHD、cGVHD分别为急、慢性GVHD；CIR：累积复发率；NRM：非复发死亡率；OS：总生存；DFS：无病生存

**表5:** 女供男组合对恶性血液病患者单倍体造血干细胞移植结局影响的多因素分析

变量	*HR*	95%*CI*	*P*值
总生存	1.056	0.708~1.576	0.788
无病生存	1.025	0.701~1.498	0.900
复发	1.058	0.591~1.510	0.810
非复发死亡	1.220	0.640~2.310	0.550
Ⅱ~Ⅳ度aGVHD	0.919	0.647~1.300	0.640
Ⅲ/Ⅳ度aGVHD	0.744	0.402~1.380	0.350
中、重度cGVHD	0.859	0.597~1.234	0.410
重度cGVHD	1.065	0.553~2.050	0.850

注：aGVHD、cGVHD分别为急、慢性移植物抗宿主病

四、供患者性别组合对≤35岁患者移植结局的影响

≤35岁患者共374例，父母供者228例（60.96％），兄弟姐妹供者123例（32.89％），子女供者4例（1.07％），非直系亲属供者19例（5.08％）。>35岁患者组274例，子女供者196例（71.53％），兄弟姐妹65例（23.72％）父母供者1例（0.37％），非直系亲属供者12例（4.38％）。

年龄≤35岁患者中，父亲供者组和母亲供者组haplo-HSCT后28 d累积中性粒细胞植入率、血小板植入率差异无统计学意义（*P*＝1.000、*P*＝0.507），Ⅱ～Ⅳ度、Ⅲ/Ⅳ度aGVHD累积发生率及移植后3年OS率、DFS、CIR、NRM率及cGVHD累积发生率差异均无统计学意义（*P*>0.05），详见表6。兄弟供者组与姐妹供者组haplo-HSCT后28 d累积中性粒细胞植入率、血小板植入率差异无统计学意义（*P*＝0.927、*P*＝0.841），Ⅱ～Ⅳ度、Ⅲ/Ⅳ度aGVHD累积发生率及移植后3年OS率、DFS、CIR、NRM率及cGVHD累积发生率差异均无统计学意义（*P*>0.05），详见表7。

**表6:** ≤35岁恶性血液病患者中父亲供者组与母亲供者组haplo-HSCT后28 d造血重建及3年主要临床结局［％，*x±s*）］

组别	例数	移植后28 d造血重建		移植后3年GVHD累积发生率				CIR	NRM	OS率	DFS率
		中性粒细胞植入	血小板植入	Ⅱ~Ⅳ度aGVHD	Ⅲ/Ⅳ度aGVHD	中/重度cGVHD	重度cGVHD				
父亲	167	99.40±0.60	96.40±1.50	34.34±3.70	16.27±2.87	35.10±4.32	6.81±2.21	22.09±3.52	8.56±2.20	75.40±3.50	69.40±3.80
母亲	61	96.72±2.42	96.7±2.60	34.43±6.15	16.39±4.78	21.33±5.95	12.01±5.26	25.71±5.84	8.23±3.56	67.70±6.40	66.10±6.20

*χ*^2^值		<0.001	0.441	<0.001	<0.001	2.794	0.476	0.649	0.052	0.570	0.309
*P*值		1.000	0.507	0.985	0.999	0.095	0.490	0.421	0.819	0.450	0.578

注：haplo-HSCT：单倍体造血干细胞移植；GVHD：移植物抗宿主病，aGVHD、cGVHD分别为急、慢性GVHD；CIR：累积复发率；NRM：非复发死亡率；OS：总生存；DFS：无病生存

**表7:** ≤35岁恶性血液病患者中兄弟供者组与姐妹供者组haplo-HSCT后28 d造血重建及3年主要临床结局［％，*x±s*）］

组别	例数	移植后28 d造血重建		移植后3年GVHD累积发生率				CIR	NRM	OS率	DFS率
		粒细胞植入	血小板植入	Ⅱ~Ⅳ度aGVHD	Ⅲ/Ⅳ度aGVHD	中/重度cGVHD	重度cGVHD				
兄弟	52	100.00±1.92	92.31±3.92	30.77±6.48	11.54±4.48	37.76±7.22	10.33±4.43	9.62±4.13	3.85±2.69	85.90±5.00	83.30±5.50
姐妹	71	100.0±1.41	94.37±2.90	40.85±5.89	18.31±4.63	52.28±6.80	26.71±5.90	19.45±5.18	5.39±3.15	77.10±5.70	75.20±5.70

*χ*^2^值		0.008	0.040	1.743	0.986	1.139	3.700	0.698	0.018	0.503	0.609
*P*值		0.927	0.841	0.187	0.321	0.286	0.054	0.403	0.895	0.478	0.435

注：haplo-HSCT：单倍体造血干细胞移植；GVHD：移植物抗宿主病，aGVHD、cGVHD分别为急、慢性GVHD；CIR：累积复发率；NRM：非复发死亡率；OS：总生存；DFS：无病生存

五、>35岁患者中供患者性别组合对移植结局的影响

年龄>35岁的患者中，儿子供者组与女儿供者组haplo-HSCT后28 d累积中性粒细胞植入率、血小板植入率差异无统计学意义（*P*＝0.517，*P*＝0.932），Ⅱ～Ⅳ度、Ⅲ/Ⅳ度aGVHD累积发生率及移植后3年OS率、DFS、CIR、NRM率及cGVHD累积发生率差异均无统计学意义（*P*>0.05），详见表8。兄弟供者组与姐妹供者组haplo-HSCT后28 d累积中性粒细胞植入率、血小板植入率差异无统计学意义（*P*＝0.271，*P*＝0.286），Ⅱ～Ⅳ度、Ⅲ/Ⅳ度aGVHD累积发生率及移植后3年OS率、DFS率、CIR、NRM、cGVHD累积发生率差异均无统计学意义（*P*>0.05），详见表9。

**表8:** >35岁恶性血液病患者中儿子供者组与女儿供者组haplo-HSCT后28 d造血重建及3年主要临床结局［％，*x±s*）］

组别	例数	移植后28 d造血重建		移植后3年GVHD累积发生率				CIR	NRM	OS率	DFS率
		中性粒细胞植入	血小板植入率	Ⅱ~Ⅳ度aGVHD	Ⅲ/Ⅳ度aGVHD	中/重度cGVHD	重度cGVHD				
儿子	124	96.72±1.69	95.90±1.90	30.33±4.18	9.02±2.60	27.41±4.54	7.86±2.72	20.44±3.88	13.78±3.45	63.90±5.10	65.80±4.70
女儿	72	95.83±2.49	91.67±3.35	25.35±5.21	7.04±3.06	40.07±6.65	6.90±3.40	10.70±3.88	14.10±4.52	77.70±5.40	75.2±5.50

*χ*^2^值		0.419	0.007	0.561	0.235	2.978	0.057	2.886	<0.001	2.120	1.784
*P*值		0.517	0.932	0.454	0.628	0.084	0.812	0.089	0.999	0.145	0.182

注：haplo-HSCT：单倍体造血干细胞移植；GVHD：移植物抗宿主病，aGVHD、cGVHD分别为急、慢性GVHD；CIR：累积复发率；NRM：非复发死亡率；OS：总生存；DFS：无病生存

**表9:** >35岁恶性血液病患者中兄弟供者组与姐妹供者组haplo-HSCT后28 d造血重建及3年主要临床结局比较［％，*x±s*）］

组别	例数	移植后28 d造血重建		移植后3年GVHD累积发生率				CIR	NRM	OS率	DFS率
		粒细胞植入	血小板植入	Ⅱ~Ⅳ度aGVHD	Ⅲ/Ⅳ度aGVHD	中/重度cGVHD	重度cGVHD				
兄弟	46	97.78±3.16	93.33±4.09	40.00±7.41	11.11±4.74	34.12±7.88	5.27±3.68	20.05±6.08	15.19±6.19	66.0±7.80	64.80±7.70
姐妹	19	100.0±5.26	89.47±7.99	42.11±11.74	5.26±5.26	22.22±10.13	11.11±7.64	5.26±5.26	10.53±7.24	82.0±9.60	84.20±8.40

*χ*^2^值		1.214	1.140	0.011	0.497	0.256	0.741	2.226	0.045	1.278	1.722
*P*值		0.271	0.286	0.917	0.481	0.613	0.390	0.136	0.832	0.258	0.189

注：haplo-HSCT：单倍体造血干细胞移植；GVHD：移植物抗宿主病，aGVHD、cGVHD分别为急、慢性GVHD；CIR：累积复发率；NRM：非复发死亡率；OS：总生存；DFS：无病生存

六、不同类型ATG预防GVHD方案对女供男、其他组移植结局的影响

共313例（48.3％）患者接受ATG-F（总量10 mg/kg）预防GVHD。女供男组和其他组haplo-HSCT后28 d累积中性粒细胞植入率差异无统计学意义（*P*＝0.221），Ⅱ～Ⅳ度、Ⅲ/Ⅳ度aGVHD累积发生率及移植后3年OS、DFS、CIR、NRM及cGVHD累积发生率差异均无统计学意义（*P*>0.05），女供男组移植后28 d累积血小板植入率显著低于其他组［（89.29±4.29）％对（94.49±1.45）％，*P*＝0.037］。详见表10。

**表10:** 含ATG-F 10 mg/kg预处理女供男组与其他组haplo-HSCT后28 d造血重建及3年主要临床结局［％，*x±s*）］

组别	例数	移植后28 d造血重建		移植后3年GVHD累积发生率				CIR	NRM	OS率	DFS率
		粒细胞植入	血小板植入	Ⅱ~Ⅳ度aGVHD	Ⅲ/Ⅳ度aGVHD	中/重度cGVHD	重度cGVHD				
女供男	56	94.64±3.09	89.29±4.29	44.44±6.85	14.82±4.88	40.83±6.98	17.50±5.37	19.73±5.40	14.33±4.74	67.60±6.30	65.90±6.40
其他	257	99.21±0.68	94.49±1.45	36.86±3.03	16.86±2.35	41.24±3.18	11.52±2.05	20.34±2.53	9.06±1.81	73.40±2.80	70.60±2.90

*χ*^2^值		1.501	4.333	1.086	0.133	0.008	1.175	0.002	0.992	0.381	0.576
*P*值		0.221	0.037	0.297	0.715	0.928	0.278	0.969	0.319	0.537	0.448

注 ATG-F：抗人T细胞兔免疫球蛋白；haplo-HSCT：单倍体造血干细胞移植；GVHD：移植物抗宿主病，aGVHD、cGVHD分别为急、慢性GVHD；CIR：累积复发率；NRM：非复发死亡率；OS：总生存；DFS：无病生存

共335例（51.7％）接受rATG-T（总量6 mg/kg）预防GVHD。女供男组和其他组haplo-HSCT后28 d累积中性粒细胞植入率、血小板植入率差异无统计学意义（*P*＝0.631，*P*＝0.404），Ⅱ～Ⅳ度、Ⅲ/Ⅳ度aGVHD累积发生率及移植后3年OS率、DFS、CIR、NRM率及cGVHD累积发生率差异差异均无统计学意义（*P*>0.05），详见表11。

**表11:** rATG-T 6 mg/kg预处理女供男组与其他供患者组合组haplo-HSCT后28 d造血重建及3年主要临床结局［％，*x±s*）］

组别	例数	移植后28 d造血重建		移植后3年GVHD累积发生率				CIR	NRM	OS率	DFS率
		粒细胞植入	血小板植入	Ⅱ~Ⅳ度aGVHD	Ⅲ/Ⅳ度aGVHD	中/重度cGVHD	重度cGVHD				
女供男	61	98.36±2.04	93.44±3.38	21.31±5.29	6.56±3.20	18.79±5.43	3.93±2.76	16.57±5.31	6.65±3.25	76.60±6.30	76.80±5.80
其他	274	99.27±0.52	95.62±1.26	31.99±2.83	10.66±1.88	31.91±4.39	12.02±3.45	18.88±3.18	9.61±1.92	74.60±3.40	71.50±3.50

*χ*^2^值		0.231	0.698	2.946	0.966	1.244	1.173	0.093	0.369	0.100	0.404
*P*值		0.631	0.404	0.086	0.326	0.265	0.279	0.760	0.543	0.752	0.525

注 rATG-T：兔抗人胸腺细胞球蛋白；haplo-HSCT：单倍体造血干细胞移植；GVHD：移植物抗宿主病，aGVHD、cGVHD分别为急、慢性GVHD；CIR：累积复发率；NRM：非复发死亡率；OS：总生存；DFS：无病生存

## 讨论

供患者性别组合是可能影响haplo-HSCT移植结局的重要因素之一[7],[10]–[16],[18]。我们先前建立了基于ATG和单纯PBSC供者来源的TCR haplo-HSCT移植体系[3]，本研究中我们首次对这一体系下就供患者性别组合对移植结局的影响进行较大规模队列研究。我们发现，不同的供患者性别组合（供患者性别相合和供患者性别不合，男供女、女供女、男供男、女供男，女供男组和其他组）均不影响haplo-HSCT的主要移植结局（OS、DFS、NRM、CIR）。经患者年龄、供者年龄、rDRI、ATG类型调整后，显示女供男组合对移植后OS、DFS、NRM、CIR无显著影响。患者年龄进一步分层后，兄弟供者/姐妹供者、儿子供者/女儿供者、父亲供者/母亲供者模式对OS、DFS、NRM、CIR等主要移植结局无显著影响。在ATG预防GVHD方案分层后，ATG-F 10 mg/kg组和rATG-T 6 mg/kg组中未观察到女供男组合对OS、DFS、NRM、CIR等主要移植结局的影响。

欧洲骨髓移植协作组基于PTCy GVHD预防方案的haplo～HSCT临床研究显示，供者性别不影响移植结局[16]。Gahrton等[25]报道对于男性患者来说，女性供者与男性供者移植获益没有差异（女供男组、男供男组中位OS期分别为18、25个月，*P*＝0.12）。Passweg等[26]亦报道供者性别不影响移植结局。国内中山大学张海燕团队报道女供男模式并不影响OS（*P*＝0.965），女性供者<30岁时女供男模式并不增加cGVHD发生率（*P*＝1.000）[27]。这些研究与本研究结论一致。然而，Stern等[28]报道供患者性别不匹配与GVHD发生率升高和更差的OS相关。Kongtim等[29]报道尽管女供男模式复发率较低，但OS率低于其他性别组合模式患者。北京大学移植团队研究结果显示女性供者与更高的NRM和更低的生存率相关[14]–[15]。与之相反的是，Berger等[17]发现在PTCy haplo-HSCT体系中母亲供者和女性供者与更低的CIR相关。以上不一致的研究结果可能与纳入研究队列的样本量、患者疾病状态、危险分层、干细胞来源、不同及预处理方案、GVHD预防方案以及移植后的治疗等差异有关。本研究rATG-T模式女供男组、其他组Ⅱ～Ⅳ度aGVHD发生率分别为（21.31±5.29）％、（31.99±2.83）％（*P*＝0.086）；Ⅲ/Ⅳ度aGVHD发生率分别为（6.56±3.20）％、（10.66±1.88）％（*P*＝0.326）；3年中重度、cGVHD发生率分别为（18.79±5.43）％、（31.91±4.39）％（*P*＝0.265），重度cGVHD发生率分别为（3.93±2.76）％、（12.02±3.45）％（*P*＝0.279）。其原因可能是rATG-T组335例患者中女供男仅61例，组间样本量差异所致的组间分析差异，结果需大样本量数据进一步验证。

不同haplo-HSCT体系中供患者亲属关系对移植结局的影响并不一致[14],[17]–[18]，患者年龄一定程度上决定亲缘供者的选择并限制了供患者关系。本研究将患者年龄分层后，进行了兄弟/姐妹、儿子/女儿、父亲/母亲供者组合对移植结局进行亚组分析，结果提示同一层亲属关系上供者性别不影响haplo-HSCT后OS和NRM。这可能与供者性别、年龄、非母系遗传抗原（NIMA）等多因素参与亲缘关系并相互影响有关[6],[14],[30]。值得一提的是，在≤35岁患者中，姐妹供者组、兄弟供者组移植后3年重度cGVHD发生率分别为（26.71±5.90）％、（10.33±4.43）％（*P*＝0.054）；在>35岁人群中，女儿供者组、儿子供者组移植后3年中重度cGVHD发生率分别为（40.07±6.65）％、（27.41±4.54）％（*P*＝0.084）；女性供者并没有呈现具有统计学差异的GVHD发生率可能受既往研究报道影响存在人为次选女供男，以及近年来预防GVHD方案及支持治疗等技术进步相关，未来需大样本的随机对照研究证实。我们还发现，女供男组与更低的累积血小板植入率相关。女供男组血小板植入率低于男供女、女供女、男供男组；进一步分为女供男和其他两组时，女供男组28 d血小板累积植入率显著低于其他组［（91.45±2.63）％对（95.08±0.95）％，*P*＝0.037］。ATG-F 10 mg/kg预防GVHD患者中，女供男组移植后28 d累积血小板植入率较低；在rATG-T 6 mg/kg预防GVHD患者中，女供男组与其他组之间移植后28 d累积血小板植入率无显著差异。目前，ATG已广泛用于GVHD预防及治疗并获得肯定的疗效[31]，不同剂量ATG的临床疗效有差异。北京大学常英军等开展的rATG-T（6 mg/kg和10 mg/kg）前瞻性随机试验结果示：总剂量10 mg/kg组的cGVHD发生率较低、感染相关死亡率较高，但是两组NRM、CIR无显著差异[32]。

rATG-T和ATG-F通过清除人外周血淋巴细胞而起到免疫抑制作用。由于免疫抗原来源和激发抗体的方法不同，两者药代动力学和药效学特征存在差异。rATG-T是人胸腺细胞免疫兔抗多克隆免疫球蛋白G（IgG）[33]，ATG-F是由人T淋巴母细胞Jurkat细胞系免疫的兔抗多克隆IgG制剂[34]，ATG-F识别抗原谱比rATG-T识别抗原谱窄，预防GVHD所需剂量ATG-F高于rATG-T。以往研究结果显示，在haplo-HSCT体系中，rATG 7.5 mg/kg与ATG-F 20 mg/kg等效[35]，ATG-F组较rATG-T组血小板植入显著延迟[36]–[37]。本研究中，ATG-F组女供男较其他组血小板植入延迟，rATG-T组女供男血小板植入未受影响。其原因可能是由于rATG-T具有针对所有阶段T细胞更为广泛的抗体谱，而ATG-F则为特异性针对活化T细胞[38]，rATG-T较ATG-F具有更强的免疫抑制作用，有利于血小板植入。然而，ATG免疫抑制作用过强也会导致免疫重建延迟[39]。能有效预防GVHD，又能更好地促进血小板植入和免疫重建的最佳ATG剂量仍需大样本、前瞻性、多中心、随机对照试验进一步探究。

本研究结果显示，在基于单纯PBSC供者来源和ATG预防GVHD的haplo-HSCT体系中，供患者性别组合不影响移植后OS、DFS、NRM和CIR等主要临床移植结局。女供男模式与延长血小板植入和更低的28 d累积血小板植入率有关。

本研究是回顾性研究，难以避免选择偏移等诸多偏倚的存在。其次，受既往研究的影响，可能存在次选女供男导致女性供者的样本量偏少影响组间统计结果；本研究并没有将非遗传性母性抗原、非遗传性父性抗原、供者特异性HLA抗体等的数据纳入研究分析；样本量限制了进一步亚组的分析，而在亚组分析中较小的样本量可能会影响统计结果造成偶然偏差。
